# Review of Afrotropical Figitinae (Figitidae, Cynipoidea, Hymenoptera) with the first records of *Neralsia* and *Lonchidia* for the region

**DOI:** 10.3897/zookeys.453.8511

**Published:** 2014-11-10

**Authors:** Simon van Noort, Matthew L. Buffington, Mattias Forshage

**Affiliations:** 1 Natural History Department, Iziko South African Museum, PO Box 61, Cape Town, 8000, South Africa; 2 Department of Biological Sciences, University of Cape Town, Private Bag, Rondebosch, 7701, South Africa; 3 Systematic Entomology Lab, USDA, c/o Smithsonian NMNH, 10th & Constitution Ave NW, Washington DC 20013; 4 Swedish Museum of Natural History, Department of Entomology, Box 50007, SE-104 05 Stockholm, Sweden

**Keywords:** Africa, Afrotropical, Cynipoidea, Figitidae, identification key, species description, taxonomy

## Abstract

The cynipoid subfamily Figitinae is poorly represented in the Afrotropical region with two genera (*Figites* Latreille and *Xyalophora* Kieffer) and six species currently known. Here we record an additional two genera (*Neralsia* Cameron and *Lonchidia* Thomson) for the region and describe three new species: *Neralsia haddocki* **sp. n.**; *Xyalophora tedjoansi* **sp. n.**; *Xyalophora tintini* **sp. n.** Benoit’s species described in 1956 are synonymized under *Figites aciculatus* (Benoit, 1956): *Figites effossus* **syn. n.**; *Figites favonius* **syn. n.**; *Figites furvus* **syn. n.**; *Figites fraudator* **syn. n.** Identification keys to the figitine genera and species occurring in the Afrotropical region are provided. Online interactive Lucid Phoenix and Lucid matrix keys are available at: http://www.waspweb.org/Cynipoidea/Keys/index.htm

## Introduction

The subfamily Figitinae is a heterogenous, and probably a paraphyletic, group of cynipoid wasps (Ronquist 1999, Buffington et al. 2007). The Afrotropical fauna is poorly known, with the first figitines from the region being described by Benoit in 1956. His treatise of the Figitidae housed in the Royal Museum of Central Africa (Tervuren), from what was then known as the Belgian Congo, included the description of four *Figites* Latreille species and a single *Xyalophora* Kieffer species all from the Democratic Republic of Congo (Benoit 1956). *Xyalophora aciculata* was subsequently transferred to *Figites* by Jiménez et al. (2008c). Since then only a single additional species, *Xyalophora provancheri* Jiménez & Pujade-Villar from Burkina Faso, has been described in Jiménez et al. 2008c). To date these are the only two figitine genera to have been recorded from the Afrotropical region.

We record two additional genera, *Lonchidia* Thomson and *Neralsia* Cameron, describe three new species, and provide identification keys to the genera and species of Afrotropical Figitinae. Benoit’s *Figites* species are synonymized with the result that *Figites*, *Lonchidia* and *Neralsia* are currently monotypic in the region.

## Materials and methods

Freshly collected specimens were point-mounted on black, acid-free cards for examination (using a Leica 205c stereomicroscope with LED light sources), photography and long-term preservation. Images were acquired using either the EntoVision multiple-focus imaging system or the Leica LAS 4.4 imaging system to illustrate diagnostic characters. The former comprised a Leica® M16 microscope with a JVC® KY-75U 3–CCD digital video camera attached that fed image data to a notebook computer. The program Cartograph® 5.6.0 was then used to merge an image series into a single in-focus image. The Leica LAS 4.4 imaging system comprised a Leica® Z16 microscope with a Leica DFC450 Camera with 0.63× video objective attached. The imaging process, using an automated Z-stepper, was managed using the Leica Application Suite V 4.4 software installed on a desktop computer. Methods for generating these photographs follow those in Buffington and van Noort (2009). Diffused lighting was achieved using techniques summarized in Buffington et al. (2005), Kerr et al. (2008) and Buffington and Gates (2009).

Morphological terminology follows that of Fontal-Cazalla et al. (2002); Ronquist and Nordlander (1989) and Jiménez et al. (2008c); cuticular surface terminology follows Harris (1979). Abbreviations and definition of measurements:

F1–F12: antennal flagellomeres 1 to 12.

T1–T8: metasomal tergites 1 to 8 (T1 = abdominal petiole).

POC (postocellar distance): shortest distance between the internal margins of the posterior ocelli.

OOC (ocello-ocular distance): shortest distance between the external margin of the lateral ocellus and the internal margin of the compound eye.

COC (ocellar distance): shortest distance between the lateral and frontal ocelli.

Relative length of the scutellar spine to length of scutellum (excluding spine) is measured in dorsal view with orientation of each surface adjusted to a horizontal plane for recording of absolute length.

Online interactive keys were produced using Lucid and Lucid Phoenix meeting the requirements of publishing both static and dynamic interactive keys under an open access model (Penev et al. 2009). All keys were produced using high quality annotated images, highlighting diagnostic characters that are integrated into the key above each couplet. This is a user-friendly output making the keys readily accessible to a wide range of users with diverse expertise. This key format circumvents the requirement of familiarity with morphological terminology associated with the particular group, because the characters are visually illustrated making the keys usable by the lay person. These keys are available at: http://www.waspweb.org/Cynipoidea/Keys/index.htm. End users can choose between three different key formats depending on their personal preference. The keys are available in three formats. Although Lucid Phoenix keys are interactive keys they are still dichotomous and a choice needs to be made at each key couplet to continue. Lucid matrix keys, on the other hand, use a different approach where relevant states from multiple character features can be selected independently until identification is achieved. For more information concerning Lucid keys visit http://www.lucidcentral.org.

All images presented in this paper are freely available through http://morphbank.net and http://www.waspweb.org using the link to individual collections.

### List of depositories

BMNH Natural History Museum, London, UK. Curator: David Notton.

CNCI Canadian National Collection of Insects, Ottawa, Canada. Curator: Andy Bennett.

MZLU Zoologiska Museet Lunds Universitet, Lund, Sweden. Curator: Christer Hansson.

RMCA Musée Royal de l’Afrique Centrale, Tervuren, Belgium. Curator: Eliane de Coninck.

SAMC Iziko South African Museum, Cape Town, South Africa. Curator: Simon van Noort.

### Figitinae

The indigenous Afrotropical genera belong to the very distinct core lineage of Figitinae, which appears to be monophyletic, and is characterised by large size, often strongly reduced wing pubescence, hairy eyes, lack of metasomal hair patch (hairy ring), and bionomics associated with attacking calyptrate Diptera in decomposing substrates. This lineage presents a very interesting morphological and life history convergence with some genera of Afrotropical Eucoilinae (e.g. *Bothrochacis*). On the contrary, the genus *Lonchidia*, of which we have so far encountered only one Afrotropical specimen of a European species, represents a separate lineage that renders the subfamily paraphyletic in phylogenetic analyses. It is easily recognizable by its confluent scutellar foveae, unusual lateral hair patches on the metasoma and sub-clavate female antennae. The recorded *Lonchidia* species may be an accidental introduction or possibly an established population of synanthropic origin.

## Systematics

### Key to Afrotropical figitine genera

**Table:** 

	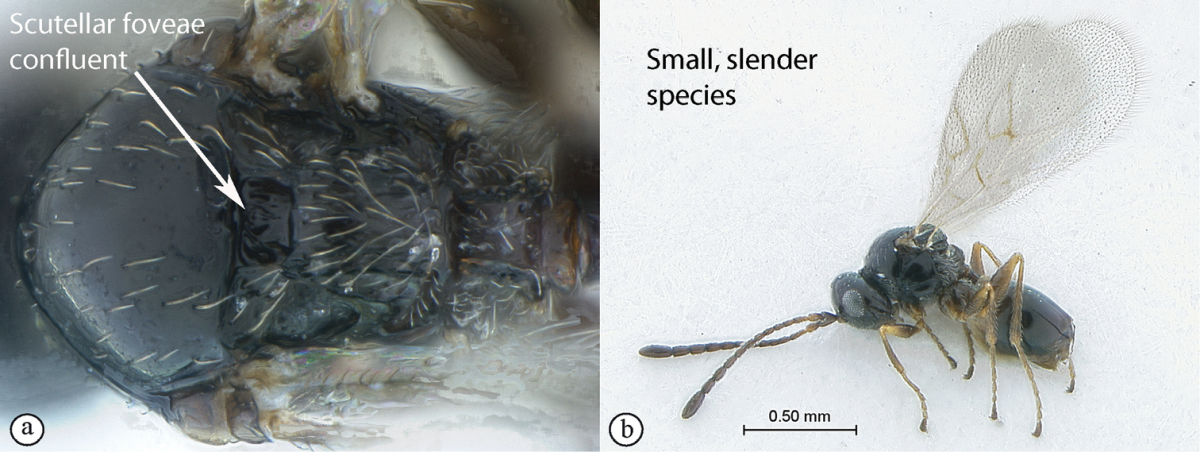	
1	Scutellar foveae confluent (a). Small, rather slender species (b)	***Lonchidia***
	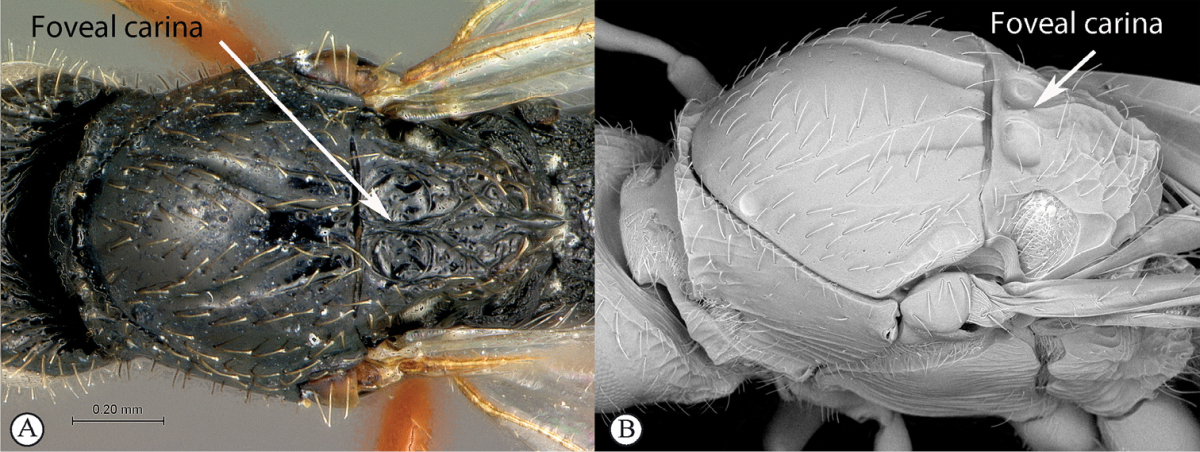	
–	Scutellar foveae distinctly separated by a median carina (A, B). Larger, strongly built species	**2**
	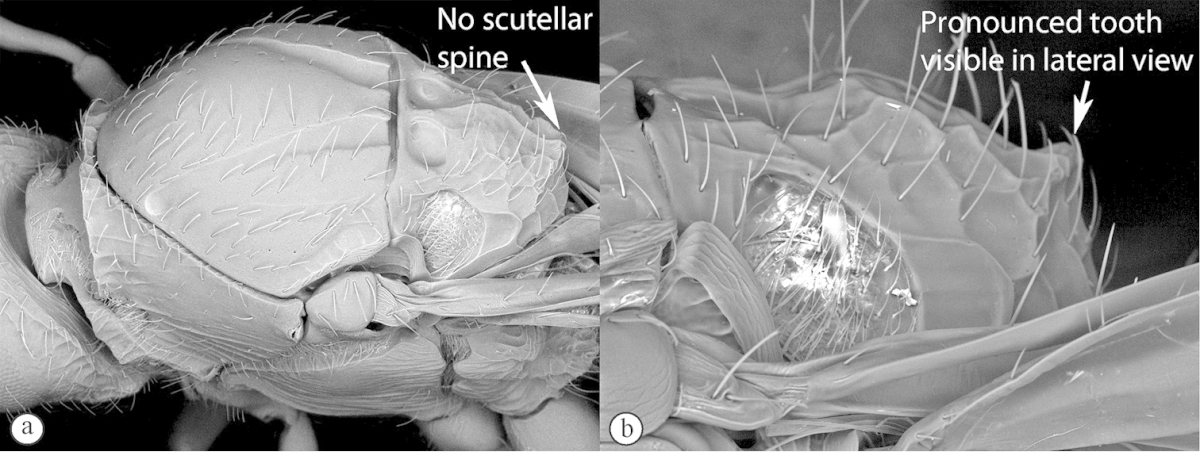	
2	No distinct scutellar spine (a), outline of scutellum in dorsal view rounded (however there is often a more or less pronounced ridge at the posteriormost point of the circumscutellar carina, which may look like a small tooth in lateral view) (b)	***Figites*** (single species currently recognised: *Figites aciculatus*)
	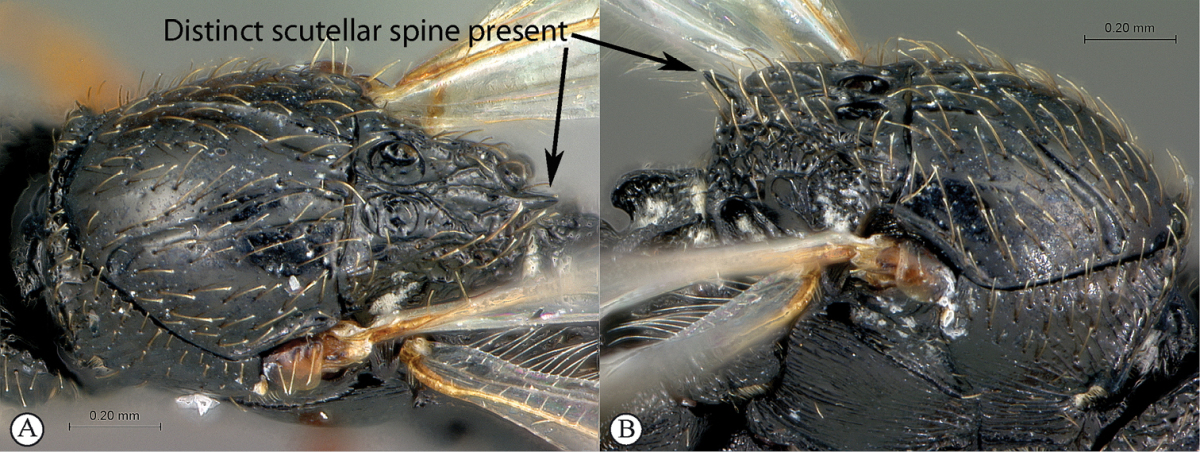	
–	Distinct scutellar spine present, obviously protruding from scutellar outline in dorsal view (A, B)	**3**
	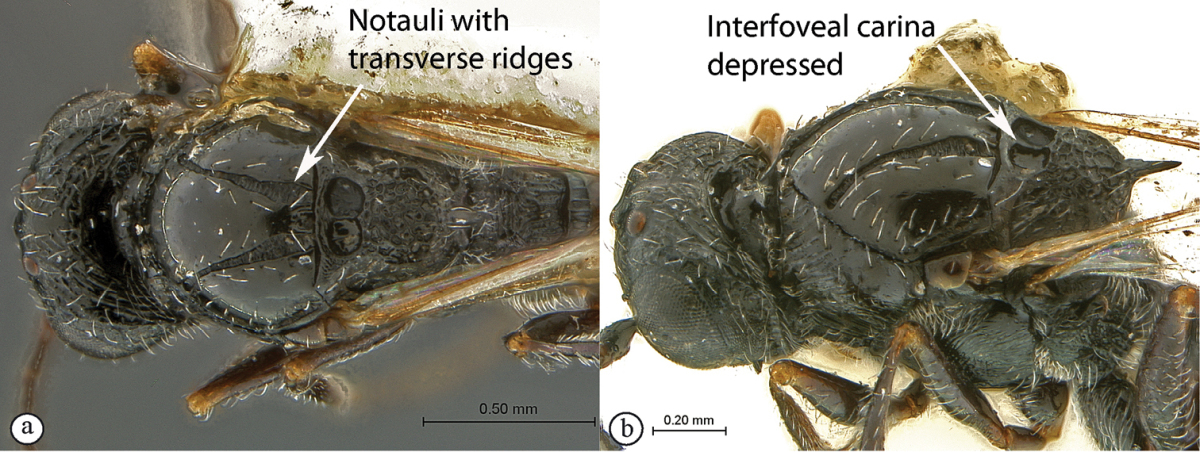	
3	Notauli sculptured with small transverse ridges (a). Interfoveal carina depressed, much lower than the level of the foveal edge (b)	***Xyalophora***
	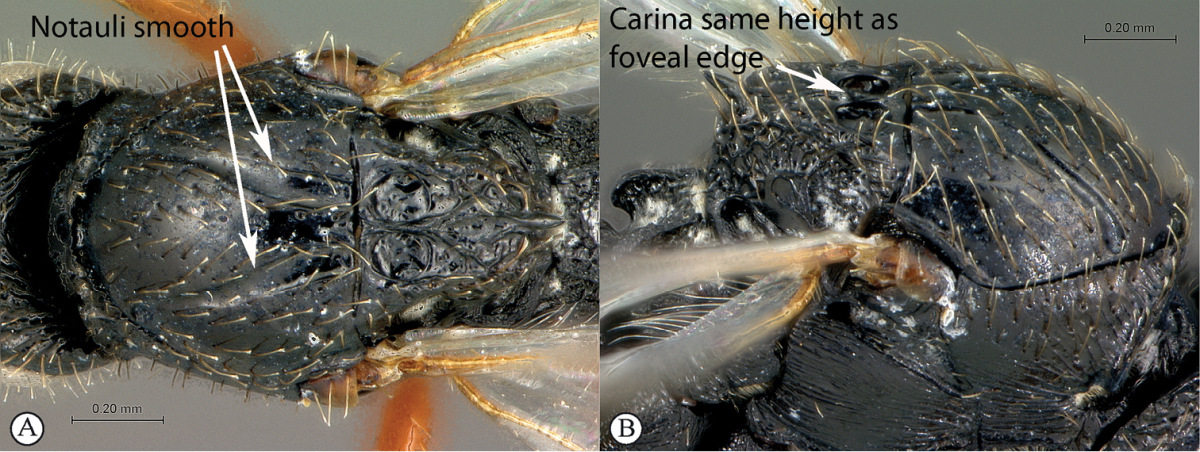	
–	Notauli smooth (A). Interfoveal carina as high as the foveal edge (B)	***Neralsia*** (single species known from Africa: *Neralsia haddocki*)

### Figites

Taxon classificationAnimaliaHymenopteraFigitidae

Latreille, 1802

Figites Latreille, 1802: 307. Type species: *Cynips scutellaris* Rossi, 1794, by subsequent designation.
Psilogaster Hartig, 1840: 187 & 202. Type species: *Figites anthomyiarium* Bouché, 1834, by original designation.
Pycnotrichia Förster, 1869: 363 & 366. Type species: *Pycnotrichia erythropa* Förster, 1869 by monotypy and original designation.
Omalaspoides Hedicke, 1913: 146. Type species: *Omalaspoides letzneri* Hedicke, 1913 by original designation.

#### Diagnosis.

Large figitines with reduced pubescence on wings (often completely hairless) and more or less striate mesosomal sides. Easily separated from *Xyalophora* and *Neralsia* by the rounded scutellum (no indication of a spine in outline in dorsal view), although the posterior scutellar rim appears as a tooth in lateral view. Stiff, stout hairs present across most of body, distally bifurcate.

#### Distribution.

Probably worldwide, but to date no records from the Oriental and Oceanic regions have been published. Afrotropical records: Democratic Republic of Congo (Benoit 1956), Cameroon, Ethiopia, Kenya, South Africa, Uganda, Yemen (new records).

#### Biology.

Parasitoids of calyptrate Brachycera larvae in decomposing substrates (Hennig 1976; James 1928; Thomas and Morgan 1972).

#### Comments.

This is a rare genus in the region. On a global scale, it is poorly circumscribed versus several smaller genera, and many of its nominal species are of doubtful identity.

### Figites aciculatus

Taxon classificationAnimaliaHymenopteraFigitidae

(Benoit, 1956)

Figures 1 , 2

Xyalophora aciculata Benoit, 1956.
Figites aciculatus (Benoit, 1956). Combination by Jiménez et al. 2008c.
Figites effossus Benoit, 1956, **syn. n.**
Figites favonius Benoit, 1956, **syn. n.**
Figites fraudator Benoit, 1956, **syn. n.**
Figites furvus Benoit, 1956, **syn. n.**
Figites aciculatus Images of all the type specimens are available on waspweb: http://www.waspweb.org/Cynipoidea/Figitidae/Figitinae/Figites/index.htm

#### Additional material examined.

**CAMEROON**, 1F: Nkoemvon, vii – viii 1979, Ms D. Jackson, *Figites* sp. det M. Forshage 2012 (BMNH); **DEMOCRATIC REPUBLIC OF CONGO**, 1M: Parc Nat. Albert, SL Edouard: r. Rwindi, 1000m, 4.ii.1936, L. Lippens (RMCA); 1M: Conge Belge: Kivu, Rutshuru, 1285m, 13 au 20.xii.1933, G.F. de Witte: 122 (BMNH); 1M: same data except:, 23 au 25.xii.1933, G.F. de Witte: 132 (RMCA); 2M: Conge Belge: P.N.A., N’Zulu (Lac Kivu), 1500m, 6 au 7.ii.1934, G.F. de Witte: 221 (BMNH; RMCA); 1M: Conge Belge: Kivu, Sake, (Lac Kivu), 1460m, 19/22.ii.1934, G.F. de Witte: 253 (RMCA); 1M: Conge Belge: P.N.A., Burunga (Mokoto) 2000m, 17 au 19.iii.1934 G.F. de Witte: 313 (RMCA); 1M: Conge Belge: P.N.A., Près Mt. Kambatembe (Forêt) 2200m, 12-iv-934, G.F. de Witte: 348 (RMCA); 2M: Conge Belge: P.N.A., Rutshuru, 1285m, 18 au 23.vi.1934, G.F. de Witte: 448 (BMNH; RMCA); 2M: Conge Belge: P.N.A., Nyarusambo, 2000m, 2.vii.1934, G.F. de Witte: 465 (RMCA); 1F: Conge Belge: P.N.A., Mt. Sesero, pres Bitashimva (Bambousi) 2000m, 1 au 2.viii.1934, G.F. de Witte: 505 (RMCA); 1M: Conge Belge: Uele, Monga, 450m, 18.iv. au 8.v.1935, G.F. de Witte: 1334 (RMCA); 1F: Conge Belge: Kivu, Rutshuru, 1285m, 29 au 31.v.1935, G.F. de Witte: 1395 (RMCA); 1F: same data except:, G.F. de Witte: 1396 (BMNH); 1F: Conge Belge: Kivu, Rutshuru, (riv. Musugereza), 1100m, 4.vii.1935, G.F. de Witte: 1607 (RMNH); 1M: Conge Belge: Kivu, Rutshuru, 1285m, 3.vii.1935, G.F. de Witte: 1610 (RMCA); 1M: same data except: G.F. de Witte: 1611 (RMCA); 2M: Conge Belge: Kivu, Rutshuru (riv. Fuku), 1250m, 5.vii.1935, G.F. de Witte: 1621 (BMNH); 1M: same data except: G.F. de Witte: 1622 (RMCA); 1F: Conge Belge: Kivu, Rutshuru, 1285m, 12.vii.1935, G.F. de Witte: 1639 (RMCA); 1F: same data except: G.F. de Witte: 1641 (BMNH); 1F: Conge Belge: Kivu, Rutshuru (Lubirizi), 1285m, 13.vii.1935, G.F. de Witte: 1645 (RMNH); 1F: Conge Belge: Kivu, Rutshuru, 1285m, vii.1935, G.F. de Witte: 1671 (RMNH); 1M: Conge Belge: Kivu, Nyongera (près Rutshuru), Butumba, 1218m, 17.vii.1935, G.F. de Witte: 1669 (BMNH); 2F: Conge Belge: Kivu, Rutshuru (riv. Rodahira), 1285m, 2.vii.1935, G.F. de Witte: 1675 (RMNH); 1F: Conge Belge: Kivu, Rutshuru (riv. Fuku), 1250m, 4.vii.1935, G.F. de Witte: 1678 (RMNH); 3F, 1M: Conge Belge: Kivu, Rutshuru 1285m, 2.vii.1935, G.F. de Witte: 1685 (RMNH); 1M: Democratic Republic of Congo Conge Belge: P.N.A., Ganza (860m), 4-6-vii-1949. Mis G.F. de Witte: 2758a (RMNH); 1F: Conge Belge: P.N.A., Secteur Tshiaberimu, Riv. Mbulikerere, affl. Dr. Talia N, 2720m, 26–28.viii.1953, P. Vanschuytbroeck & V. Hendrickx, 4999-5005 (BMNH); 2 F 2M: Conge Belge: P.N.A., Mont Hoyo, 1280m, sur plantes basses, 7–15.vii.1955, P. Vanschuytbroeck, 13274-309 (BMNH; RMNH); 1F: Conge Belge: P.N.A., 21-iv-1955, P. Vanschuytbroeck & R. Fonteyn, 12.813-16 Secteur Tshiaberimu, Mont Musienene, 2.680 m, près de Kirungu (RMNH); 1M: Conge Belge: P.N.A., 16-vii-1957, P. Vanschuytbroeck VS 84 (2) Secteur Nord, riv. Lesse, affl. G. Semliki, 695 m (RMNH); **ETHIOPIA**, 1M: Nazareth, 6700’, 16–19.II.62, S.M. Clark (CNCI); **KENYA**, 2F: Kakamega Forest, 18.xii.1970, A.E. Stubbs, B.M. 1972-211 (BMNH); **SOUTH AFRICA**, 1F: Eastern Cape Province, Port St John, Pondoland, June 12–30 1923, R.E. Turner, Brit. Mus. 1923-363 (BMNH); 1F: [Kwazulu-Natal], Natal, Van Reenen, Drakensberg 1–22.i.1927, R.E. Turner, Brit. Mus. 1927-54 (BMNH); 1F: Eastern Cape Province, Katberg, 15–30.i.1933, R.E. Turner, Brit. Mus. 1933-108 (BMNH); **UGANDA**, 1M: Mulange, R, Dummer, Nov, 1922. *Figites* det M. Soderlund 1993, SAM-HYM-P002880 (SAMC). 1F: Kazhara, H.C. Taylor, iii.1939, Brit. Mus. 1956-25, *Figites* det. J. Quinlan, 1957 (BMNH); 1 F: Kawanda, T.H.C. Taylor, xi.1942, Brit. Mus. 1956-25, *Figites* det. J. Quinlan, 1957 (BMNH); 2M: Ruwenzori Range, Namwamba Valley, 10 100 ft., T.H.E. Jackson, xii.1934-i.1935, B.M. E. Africa. Exp., B.M. 1935-203 (BMNH); **YEMEN**, 1F: Ar Rujum, 15°27.47'N, 43°38.10'E, 16.10.00-15.01.01, in Malaise-trap, coll. A. van Harten & A.M. Hager, 5464, SAM-HYM-P046196 (SAMC).

#### Distribution.

Democratic Republic of Congo (Benoit 1956), Cameroon, Ethiopia, Kenya, South Africa, Uganda, Yemen (new records).

#### Description synopsis with overview of morphological variation.

*Female*. Head, mesosoma, metasomal tergite 1, coxae black; rest of metasoma reddish brown; scape black, rest of antennae pale to dark reddish brown, multiporous fig sensilla (MPS) concolourous with segment or more silver, legs testaceous, femora darker. Antennae pale to dark reddish-brown, 11 flagellar segments; flagellar segment 1 longer than segment 2; flagellar segments 1–4 with no MPS; remaining 7 segments with single row of MPS except for the club segment which has 2 rows; club segment about twice the length of penultimate segment and 1.5× longer than wide. Occiput with reticulate sculpturing or parallel carina, but may be fairly smooth with only a few weak parallel carina on posterior edge of vertex orientated parallel to genal carina. Pronotal fig cordate, smooth. Medial posterior impression present or absent between notauli. Scutellar posterior rim raised into what appears to be a tooth visible in lateral view. Mesopleuron completely striate or with smooth medial patch. Marginal cell open or closed. Marginal vein often present, but hyaline and only pigmented for basal half to three-quarters of vein. Cell usually less than twice as long as wide 1.3×–1.8× but may be 2.0×. Basal vein usually shorter (0.65–0.85×) than portion of subcostal vein forming 1^st^ cubital cell, but may be longer (1.25×). Propodeal shelf as long as metasomal petiole in lateral view. Metasomal tergite 2 smooth or with longitudinal striations that can form a short collar or extend almost the length of tergite. Tergite 3 smooth or sometimes with lateral striate patch, striations often weak. Fore tarsal basal segment = the remaining segments in length.

**Figure 1.**
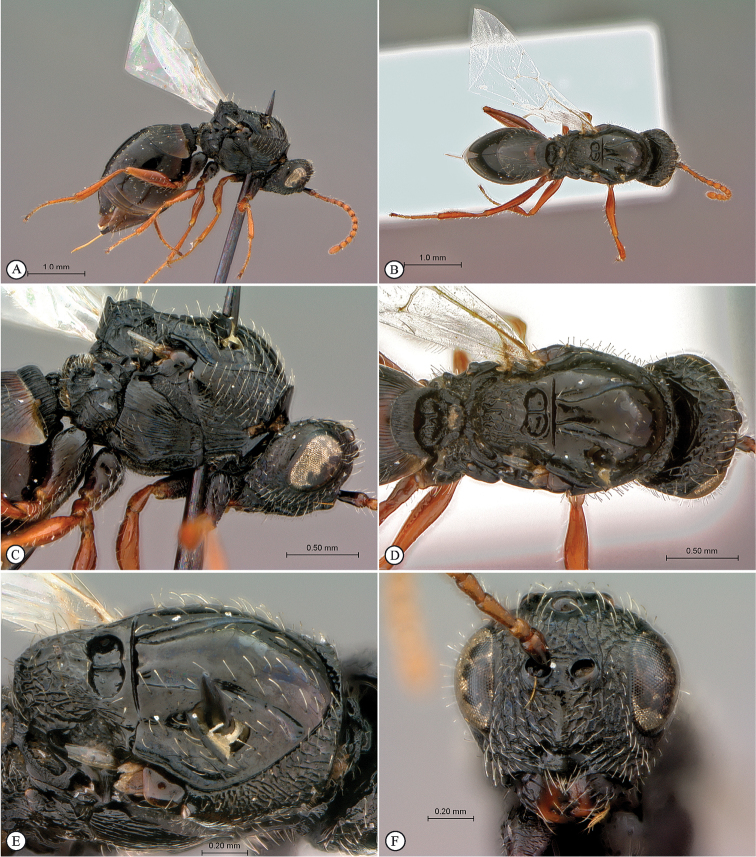
*Figites aciculatus* (Benoit), holotype female. **A** lateral habitus **B** dorsal habitus **C** head and mesosoma, lateral view **D** head and mesosoma, dorsal view **E** mesosoma, dorso-lateral view **F** head, anterior view.

*Male*. Colour as in female. Twelve flagellomeres. First two flagellar segments equal in length and each equal to scape & pedicel combined. 1st flagellar segment 3× longer than wide. Scape 3× pedicel length. Face reticulate to centrally smooth with weak lateral carina. Occiput reticulate to rather smooth, with faint indications of carinae in reticulate pattern. Toruli separated by a third to a half of their own diameter. Eyes separated by just over 1.1× eye length. Pronotal collar smooth. Mesopleuron with dorsal medial smooth patch. Slight medial posterior depression between notauli. Scutellar tooth strong in lateral view or may be almost absent with scutellar rim very low in specimens that are generally less sculptured. Vertical parallel carina on scutellar posterior vertical surface. Pronotal shelf same length as metasomal tergite 1 petiole in lateral view. Pronotum with two parallel raised longitudinal carinae bounding medial rectangular area. Marginal cell may be obviously closed with pigmented vein or may be open with vein loosing pigment. 1.5×–2.0× longer than wide. Basal vein usually shorter (0.7–0.9×) than portion of subcostal vein forming 1^st^ cubital cell. Tergite 2 smooth or with very faint weak striations near base. Tergite 3 may have a very small patch of very weak striations.

**Figure 2.**
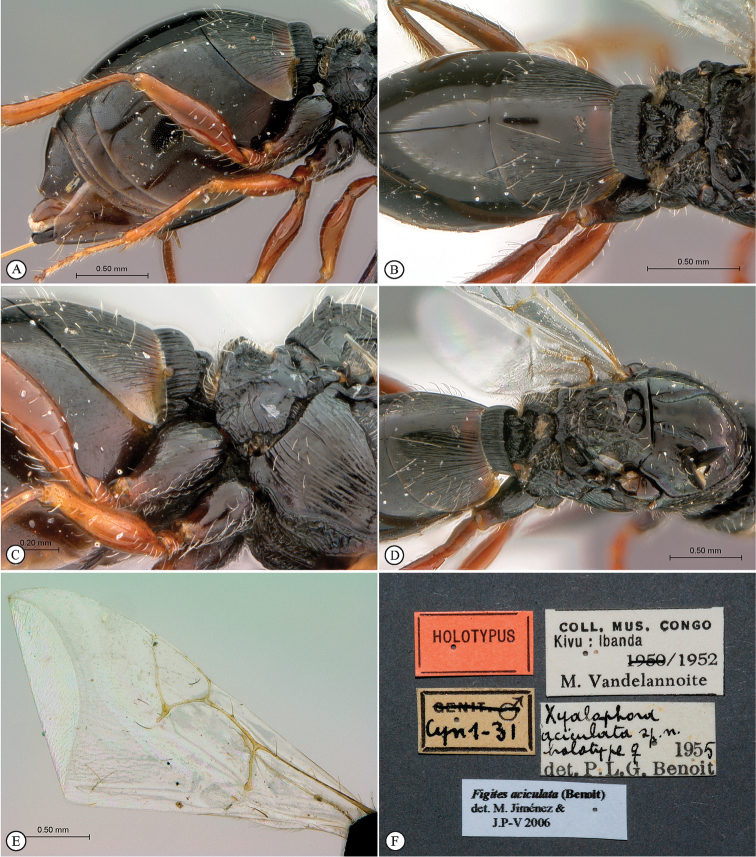
*Figites aciculatus* (Benoit), holotype female. **A** metasoma, lateral view **B** propodeum and metasoma, dorsal view **C** propodeum and partial metasoma, lateral view **D** mesosoma and partial metasoma, dorso-lateral view **E** forewing **F** data labels.

#### Comments.

Benoit described five species (in two genera) based on single specimens using differential characters that are highly variable and likely to be indicative of intraspecific variation. Benoit deemed that *Figites aciculatus* possessed a scutellar spine and hence placed this species in *Xyalophora*. Examination of the holotype clearly shows this specimen to possess the same character state as in the rest of the *Figites* species that he described, i.e. a rounded scutellum with no indication of a spine in outline in dorsal view (distinguishing this genus from both *Xyalophora* and *Neralsia*), although the posterior scutellar rim appears as a small tooth in lateral view. Jiménez and Pujade-Villar (2008c) correctly transferred this species to *Figites*. Although we initially attempted to find correlating characters across an examined series of 57 specimens to corroborate Benoit’s species delimitation, there was no consistency in reliably diagnostic character states, alone or in combination. In particular, the degree of closure of the radial vein, and degree of sculpturing, including presence (and extent of) or absence of striations on metasomal tergites 1 and 2, which are two of the main characters used by Benoit to define his species, are variable across the series of specimens that we have examined. Specimens cannot always reliably be placed in one or the other of Benoit’s species, because of possession of different character state combinations and a continuous range of variation across body size (specimens range in body length from 2–4 mm). The degree and extent of sculpturing varies with specimen size with smaller specimens tending to be smoother overall with reduced sculpturing on the occiput, pronotum, mesopleuron, metacoxae and metasomal tergites. A potential useful character state is the type of sculpturing present on the occiput, which may be reticulate or have the cross-carina absent creating parallel carina. Although appearing very different, this latter character state is likely to be related to a reduction in sculpturing and is not consistently correlated with other potentially diagnostic characters. Larger specimens tend to be more sculptured with reticulate occipital sculpturing and a closed marginal cell and smaller specimens less sculptured with parallel carina on the occiput and an open marginal cell, but there are intermediates and exceptions. Many of the specimens with occipital reticulate sculpturing and a closed marginal vein could be assigned to *Figites aciculatus* (the holotype is a large specimen with a 4 mm body length), but there were also specimens with a closed marginal cell and parallel carina. There are even specimens that have different degrees of closure of the marginal cell on each wing, so clearly this is a plastic character e.g. a female from Kivu, Rutshuru (Democratic Republic of Congo) has one wing with the marginal cell open and other wing with the marginal cell closed. In many specimens the vein is often present along the entire wing margin, but not completely pigmented and therefore depending on lighting, background colour and magnification strength, the marginal cell can be interpreted as open or closed, compounding reliable identification. The relative dimensions of the marginal cell also exhibit a range of variability (1.6–2.1× as long as wide), and the basal vein is usually much shorter (0.67–0.71×) than the portion of the subcostal vein forming the 1^st^ cubital cell. However, in the Cameroon specimen, the marginal cell can be a little more than twice as long as wide and the basal vein is longer (1.25×) than the portion of subcostal vein forming the 1^st^ cubital cell. Together with an extended hypopygium, this specimen could represent an undescribed species, but it appears to fit at the end of the range of variability of these characters. The hypopygium usually does not extend beyond the end of the metasoma, but depending on the extension or contraction of the metasomal segments the hypopygium (and ovipositor sheaths) may extend beyond the end of the metasoma, as in the Cameroon specimen.

The degree of striation on the pronotum is also variable with the posterior medial lateral section usually smooth and this smooth area can extend to the anterior medial margin. The mesopleuron can be completely striate or with a smooth medial patch, the latter state more typical of males. Two other characters used by Benoit are also variable and difficult to characterize: the medial depression, sometimes present posteriorly between the notauli, is highly variable in depth and presence and difficult to discern if weakly present; the presence or absence of the forewing areolet is also variable, usually very difficult to discern and arguably closed or open if it is visible.

Based on re-interpretation and subsequent appreciation that the diagnostic characters used by Benoit are highly plastic, in combination with the fact that his species concepts are based on single specimens that are representative of different points in a continuous range of variation, we synonymize these taxa under *Figites aciculatus*. We chose this name because the type is in good condition and is representative of the most common morphology (within the range of intra-specific variation) exhibited by the specimens we have examined.

### Lonchidia

Taxon classificationAnimaliaHymenopteraFigitidae

Thomson, 1862

Lonchidia Thomson, 1862: 413. Type-species: *Figites maculipennis* Dahlbom, 1842, by subsequent designation.

#### Diagnosis.

Small, rather slender, and more or less strongly pubescent figitines, easily recognised by the confluent scutellar foveae. Pubescence is dense on patches on the sides of the large metasomal tergite, as a collar on the pronotum, on the propodeum, and rather dense also on metapleura and metacoxae. The marginal cell of the forewing is characteristically short, and the antennae in females end with an enlarged apical flagellomere.

#### Distribution.

Mostly a Holarctic genus, here reported for the first time from the Afrotropical region. Afrotropical records: South Africa.

#### Biology.

No host records exist. Hosts are expected to be saprophagous Brachycera larvae, but these wasps appear less directly associated with decomposing substrates like dung and carrion than many other figitines. Species in North America are frequently collected in pasture land or meadow, in close approximation to domesticated bovines (Buffington, pers. obs.)

#### Comments.

The only Afrotropical specimen seen so far is from South Africa and may be an accidental introduction. It corresponds to a form present in Europe, which is currently considered as belonging to *Lonchidia clavicornis* Thomson, but which differs from the type specimen in some minor respects. Further studies may possibly show that this is a separate, currently unnamed, species.

### Lonchidia clavicornis

Taxon classificationAnimaliaHymenopteraFigitidae

Thomson, 1862

Figure 3

#### Material examined.

1F: South Africa, Cape Province, 10 km S of Citrusdal, Koornlandskloof, S32°40', E19°02', 5–9.X.1994, marshy meadow at riverside, Malaise trap, leg. Michael Söderlund, MZLU 2013 227 (MZLU).

### Neralsia

Taxon classificationAnimaliaHymenopteraFigitidae

Cameron, 1883

Neralsia Cameron, 1883: 4. Type species: *Neralsia rufipes* Cameron, 1883, by monotypy and original designation.
Xyalosema Dalla Torre & Kieffer, 1910: 73 & 94. Replacement name for *Solenaspis* Ashmead, 1887.
Solenaspis Ashmead, 1887: 151 & 155. Unavailable junior homonym of *Solenaspis* Osten Sacken, 1881 (Diptera). Type species: *Solenaspis hyalinipennis* Ashmead, 1887 by monotypy and original designation.

#### Diagnosis.

Along with *Xyalophora*, this is the only known figitine in the Afrotropical Region with a scutellar spine. *Neralsia* can be distinguished from *Xyalophora* by whether or not the notauli are horizontally striate: smooth in *Neralsia*, striate in *Xyalophora* (Jiménez et al. 2008c). Also, most *Neralsia* have longer, more robust scutellar spines than *Xyalophora*, but in specimens we have examined, this character varies with overall size of the specimen. This taxon also resembles *Prosaspicera* (Aspicerinae), which also possess a distinct scutellar spine, but can be separated from *Prosaspicera* by the lack of a facial impression on the head (present in *Prosaspicera*), and lack of a ligulate metasoma T2.

**Figure 3.**
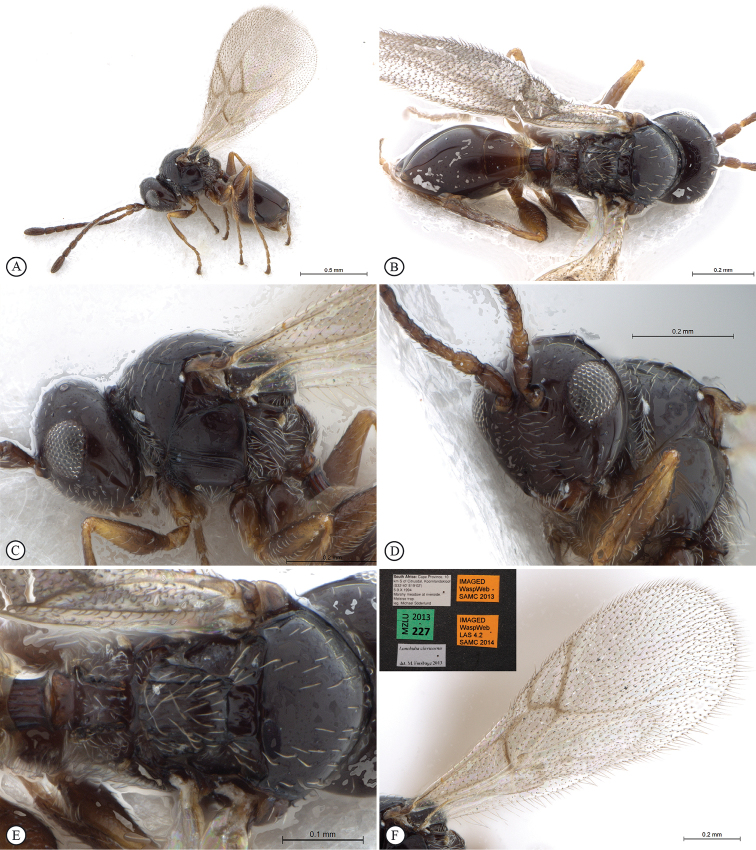
*Lonchidia clavicornis* Thomson female. **A** lateral habitus **B** dorsal habitus **C** head and mesosoma, lateral view **D** head anterio-lateral view **E** mesosoma, dorsal view **F** wings (inset: data labels).

#### Comments.

Rare in Afrotropical region. The genus is extremely species-rich in the Neotropical region and has recently been revised in a series of papers by Jiménez et al. (2004, 2005a, 2005b, 2006, 2007, 2008a, 2008b), Jiménez and Pujade-Villar (2009); Petersen-Silva and Pujade-Villar (2010); Petersen-Silva et al. (2010) and Pujade-Villar et al. 2006. *Neralsia* is also common throughout the Nearctic Region, but species limits have not been thoroughly established (Buffington, pers. obs.)

#### Distribution.

Mainly Neotropical, but with single species in the Nearctic and Afrotropical regions. Purported records from the Oriental region and the east Palearctic are unconfirmed. Afrotropical records: Central African Republic, South Africa (here).

#### Biology.

Parasitoids of calyptrate Brachycera larvae in decomposing substrates (Díaz et al. 2000; Thomas and Morgan 1972).

### Neralsia haddocki

Taxon classificationAnimaliaHymenopteraFigitidae

van Noort, Buffington & Forshage sp. n.

http://zoobank.org/6D429A4D-D056-4BBB-918E-6DF68DBD2447

Figures 4 , 5 , 6

#### Type material.

**HOLOTYPE.** Female: **CENTRAL AFRICAN REPUBLIC**, Prefecture Sangha-Mbaéré, Réserve Spéciale de Forêt Dense de Dzanga-Sangha, 12.7km 326° NW Bayanga, 3°00.27'N, 16°11.55'E, 420m, 16–17.v.2001, S. van Noort, Malaise trap, CAR01-M145, Lowland Rainforest; SAM-HYM-P025026 (SAMC). **Paratype.** 1M: **SOUTH AFRICA**, [Eastern Cape Province], Port St John, Pondoland, 16–28.iv.1924, R.E. Turner, Brit. Mus. 1924-235 (BMNH).

#### Distribution.

Central African Republic, South Africa.

#### Etymology.

The specific epithet *haddocki* is in the genitive case and is for Captain Haddock, the comic book character by Hergé. The derivation has specific reference to Captain Haddock’s consistent state of inebriation and utterance of the phrases “ten thousand thundering typhoons” and “billions of bilious blue blistering barnacles”, expletives commiserate with the discovery and generic determination of this novel Afrotropical record in the CAR ethanol samples.

#### Diagnosis.

*Neralsia haddocki* can be separated from all other described world *Neralsia* species by the closed marginal cell. A defined true vein is present along the wing margin completely closing the marginal cell. A number of the Central American and West Indies species have a darkening on the wing margin, but this is not considered to be a true vein (Jiménez et al. 2008b). The carina present between the scutellar fovea is at the same height as the outer foveal edge.

#### Description.

FEMALE. Length 3.2 mm. Head, mesosoma, coxae, antennal scapes, and metasoma T1 black; rest of metasoma dark brown to reddish-brown; antennae gradually lightening from the dark-brown pedicel to the light reddish-brown F8-F11; legs reddish-brown. Wings transparent, without any infuscation.

*Head*. Head subquadrate, slightly wider 1.05× than long excluding mandibles. Entire head, including eyes and mandibles, with scattered strong white pubescence. Eyes slightly bulging, projecting beyond outer margin of gena in frontal view. Antenna 13 segmented; F1 marginally longer (1.07×) than F2; flagellum widening toward apex. Vertex polished, setose; ocellar fig slightly raised, polished, setose; lateral ocellus diameter 0.93× the distance between lateral and median ocellus (COC); POC:OOC:COC = 30:20:15. Upper face with reticulate carina radiating away from outer edges of toruli towards ocelli; antennal scrobes not delimited, but inner scrobal area with parallel finer carina arcing dorsally between toruli; semi-circular polished area anterior to medial ocellus. Occiput weakly concave in dorsal view, rugulose, with some parallel carina, medially polished. Lower face rugulose, with carinae directed towards middle of face; face humped between toruli and clypeal margin, protruding in lateral view; toruli projected on shelf. Upper clypeal margin defined by two pronounced lateral excavations, each encompassing an anterior tentorial pit. Clypeus with strong medial convexity, concave ventrally with strong, pubescence; clypeal margin evenly convex. Gena finely punctate proximal to eye, rugose towards mandible and strong genal carina.

**Figure 4.**
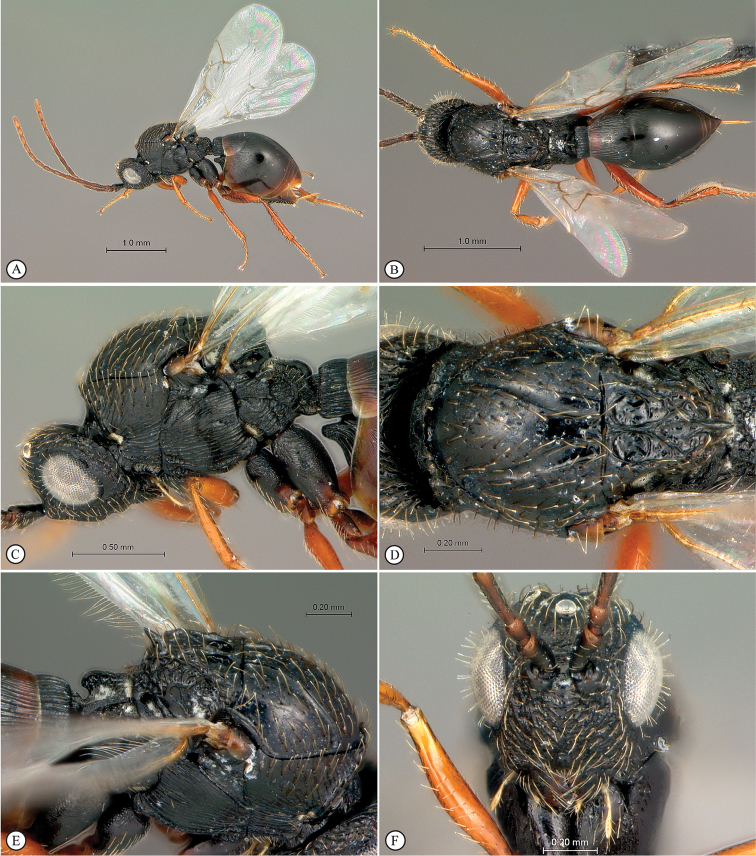
*Neralsia haddocki* sp. n., holotype female. **A** lateral habitus **B** dorsal habitus **C** head and mesosoma, lateral view **D** head and mesosoma, dorsal view **E** mesosoma, lateral view **F** head, anterior view.

*Mesosoma*. With scattered strong golden pubescence dorsally, laterally glabrous. Anterior fig of pronotum smooth dorso-medially with vertical parallel carina ventrally and laterally; fig dorsally and laterally defined by strong pronotal carina, which is medially indented. Lateral surface of pronotum horizontally striate, striations radiating away from submedial pronotal depression, containing dense, white fluff; pronotum ventrally with patch of dense white setae. Mesoscutum polished. Notauli almost complete, terminating just before anterior margin of mesocutum; smooth; posteriorly broadening; median mesoscutal impression present as small insignificant depression; parascutal impressions weakly defined, smooth. Mesoscutum convex, scutellum flat. Scutellar fovea not subdivided by longitudinal carinae. Foveal carina at height of outer foveal edge. Scutellum laterally areolate-rugose, medially polished with reticulate carinae. Scutellar spine short, 0.3× scutellar length (excluding spine). Mesopleural triangle defined with weak ventral carina, horizontally striate with very fine pubescence; mesopleuron horizontally striate, striations denser dorsally than ventrally; mesopleural carina present, defined dorsally by parallel impression. Mesopleural pit present. Metepisternum antero-ventrally excavated with pubescence, medially rugulose. Metepimeron depressed with pubescence. Lateral propodeal carina very prominent, thick and strongly raised, dorsal margin convex in lateral view. Median and lateral propodeal areas rugulose. Lateral propodeal area with strong pubescence. Calyptra prominent, strongly raised. Rs+M and areolet of forewing weakly defined. Basalis vein present. M+Cu1 weakly defined. Marginal cell 1.8× as long as wide, closed along wing margin. Margin with fringe of setae. Coxae sculpture, rest of legs polished, pubescent. Mesotibial spurs subequal in length; metatibial outer spur shorter than inner spur. Ratio of first metatibial segment to the remaining 4 segments: 0.88×.

*Metasoma*. Tergite 3 strongly striate; tergite 4 anteriorly striate grading into punctate laterally and posteriorly; dorso-medially polished; remaining tergites finely punctate. Abdominal petiole (T2) strong, longitudinally striate, 2.2× as wide as long in dorsal view. T4 the largest tergite. Relative dorsal length of T3–T8: 60:100:8:8:15:40. Ovipositor valves not extending beyond apex of metasoma, enclosed within hypopygium. Ovipositor clip present, elongate with well-developed ventral lobe. Hypopygium with fringe of long setae running down each side; not extending beyond T8.

**Figure 5.**
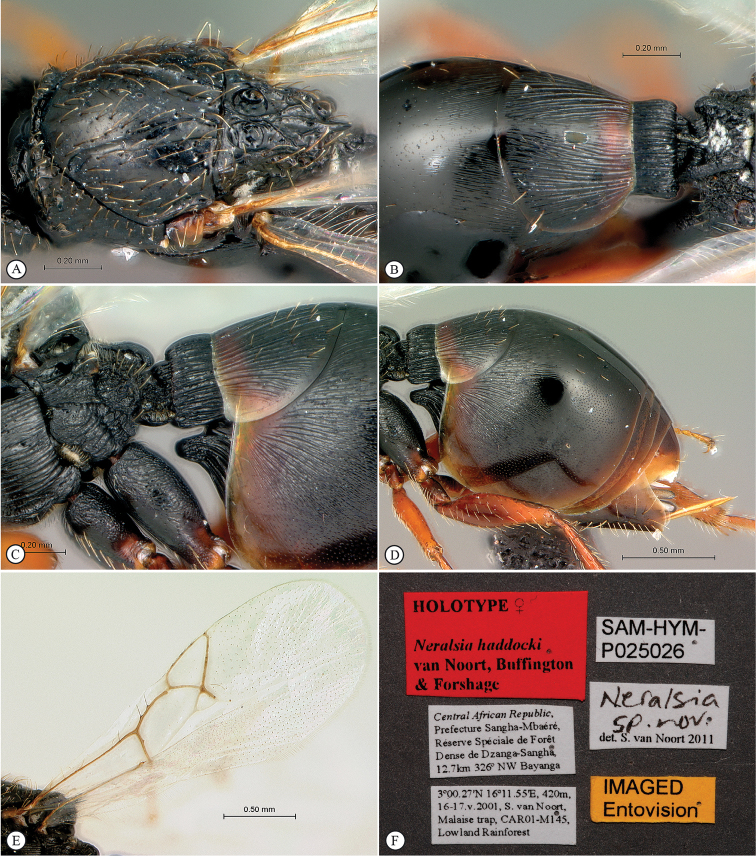
*Neralsia haddocki* sp. n., holotype female. **A** mesosoma, dorso-lateral view **B** propodeum and partial metasoma, dorsal view **C** propodeum and partial metasoma, lateral view **D** metasoma, lateral view **E** forewing and hindwing **F** data labels.

MALE. Length 2 mm. Head, mesosoma, coxae, antennal scapes, and posterior two-thirds of metasoma T1 black; rest of metasoma and coxae dark reddish-brown; antennae light reddish-brown; rest of legs pale yellowish-brown except for hind femur, which is reddish-brown for proximal two-thirds. Wings transparent, without any infuscation.

*Head*. Head more transversely globose than in female, 1.2× wider than long excluding mandibles. Entire head, including eyes and mandibles, with scattered strong white pubescence. Eyes strongly bulging, projecting well beyond outer margin of gena in frontal view. Antenna 14 segmented; F1 same length as F2; flagellar segments gradually shortening towards apex; except for long ultimate segment (1.15× length of F1). Vertex granulate, setose; ocellar fig slightly raised, polished, setose; lateral ocellus diameter 1.2× the distance between lateral and median ocellus (COC). Upper face with reticulate carina radiating away from outer edges of toruli towards ocelli. Occiput weakly concave in dorsal view, with parallel semi-reticulate carina. Face and clypeus as in female.

*Mesosoma*. With scattered strong white pubescence dorsally, laterally glabrous, otherwise mesosoma as in female. Forewing more setose than in female.

*Metasoma*. Tergite 3 polished; tergite 4 anteriorly polished grading into punctate laterally and posteriorly; remaining tergites finely punctate. Abdominal petiole (T2) strong, longitudinally striate, twice as wide as long in dorsal view. T4 the largest tergite. Relative dorsal length of T3–T8: 10:14:1:1:2:1.

**Figure 6.**
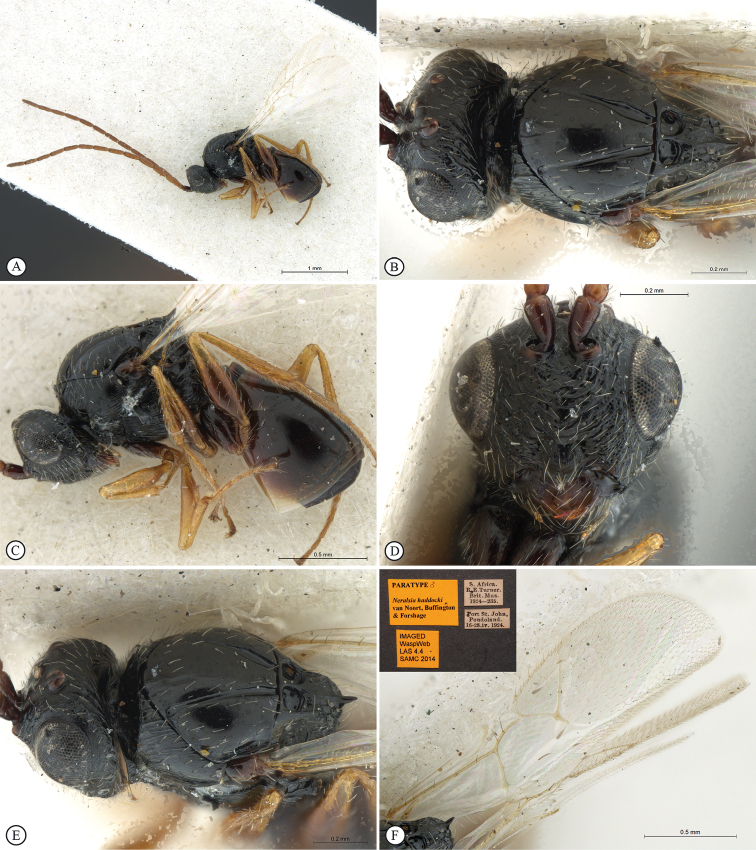
*Neralsia haddocki* sp. n., paratype male. **A** lateral habitus **B** head and mesosoma, dorsal view **C** body, lateral view **D** head anterior view **E** head and mesosoma, dorso-lateral view **F** wings (inset: data labels).

### Xyalophora

Taxon classificationAnimaliaHymenopteraFigitidae

Kieffer, 1901

Xyalophora Kieffer, 1901: 344. Type species: *Figites clavatus* Giraud, 1860, by monotypy and original designation.
Ceraspidia Belizin, 1952: 301. Type species: *Ceraspidia japonica* Belizin, 1952, by monotypy and original designation.

#### Diagnosis.

*Xyalophora* shares the presence of a scutellar spine with *Neralsia*, absent in *Figites* and *Lonchidia*. *Xyalophora* can be separated from *Neralsia* by the presence of transversely striate notauli (smooth in *Neralsia*), and an often slightly smaller scutellar spine; this second character, however, is often linked to adult body size and should be used with caution. As in the case of *Neralsia*, species of *Xyalophora* can be superficially similar to *Prosaspicera* (Aspicerinae), but can be separated from that taxon by the lack of a facial impression on the head, as well as the lack of a ligulate metasomal T2. All three African species have the occipital carinae directed towards the ocellar area and separated in the middle by a smooth surface as well as a smooth interocellar area.

#### Distribution.

Probably worldwide, but no records from the Oriental region are published. Afrotropical records: Burkina Faso (Jiménez et al. 2008); Democratic Republic of Congo, Mali, Namibia, South Africa (here).

#### Biology.

Parasitoids of calyptrate Brachycera larvae in decomposing substrates (Ionescu 1969).

#### Comments.

A rare genus that has been recently revised by Jiménez et al. 2008.

### Species richness

*Xyalophora provancheri* Jiménez & Pujade-Villar, 2008 (Burkina Faso)

*Xyalophora tedjoansi***sp. n.** (Mali)

*Xyalophora tintini***sp. n.** (Democratic Republic of Congo, Namibia, South Africa)

#### Key to Afrotropical species of *Xyalophora*

**Table:** 

	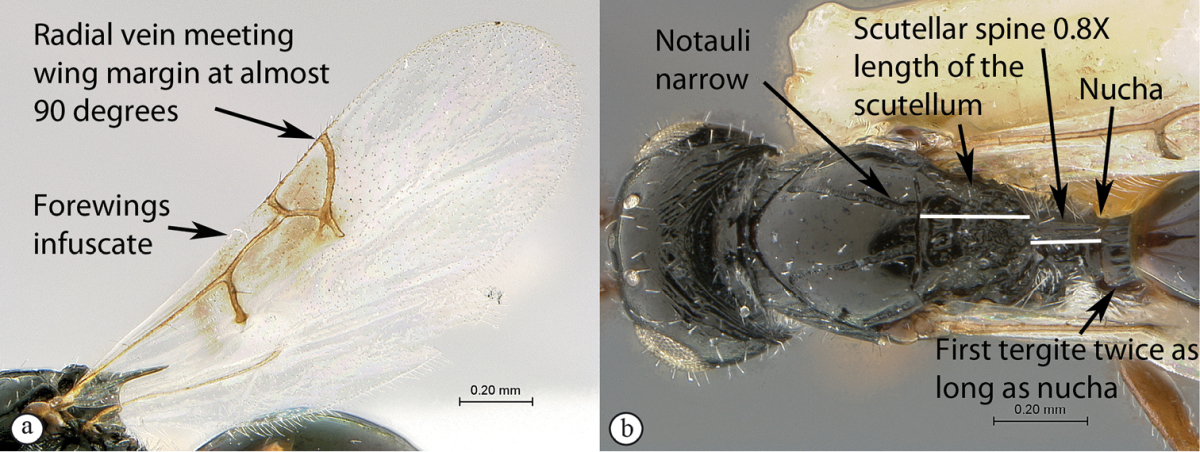	
1	Forewings infuscate over area surrounding venation (a). Marginal cell 1.55× as long as wide (a), venation thick with a very thin marginal vein (a); radial vein meeting wing margin at almost 90 degrees (a). Scutellar spine long, 0.8× length of the scutellum (excluding spine) (b). Notauli narrow (maximum width 0.35× the minimum distance separating notauli towards posterior mesoscutal margin) (b). Head subquadrate, 1.1× wider than long. First tergite (petiole) long (0.6× as long as high in lateral view; twice as long as nucha in dorsal view) (b)	***Xyalophora tedjoansi* sp. n.**
	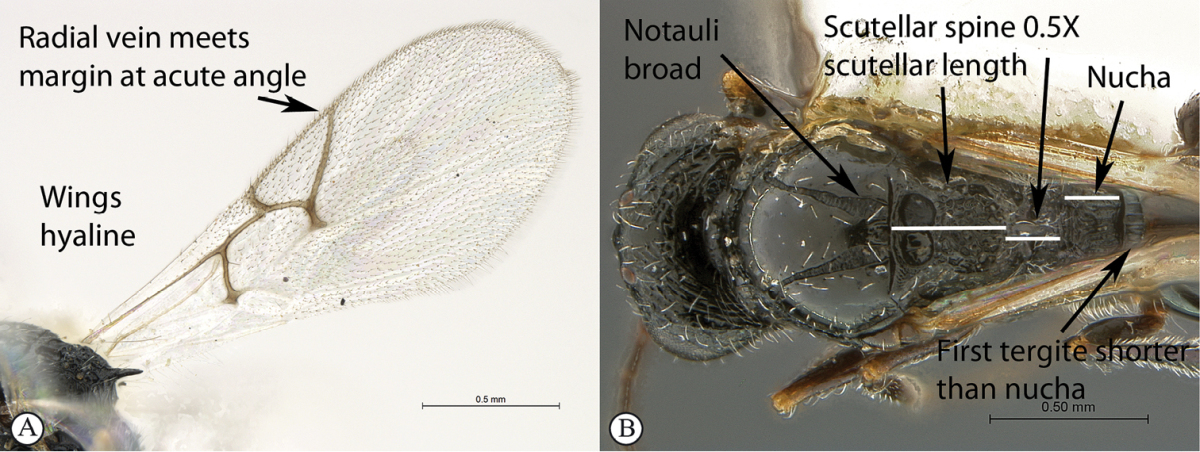	
–	Forewings hyaline (A). Marginal cell 2–2.5× as long as wide (A), venation thinner with less contrast in thickness with marginal vein (A); radial vein meets wing margin at acute angle (A). Scutellar spine shorter, 0.5× the length of the scutellum (excluding spine) (B). Notauli widened posteriorly (maximum width 0.7–0.8× the minimum distance separating notauli towards posterior mesoscutal margin) (B). Head distinctly (1.25×) wider than long. First tergite short (0.2–0.25× as long as high in lateral view; either a third of nucha length (B) or equivalent in length to nucha in dorsal view)	**2**
	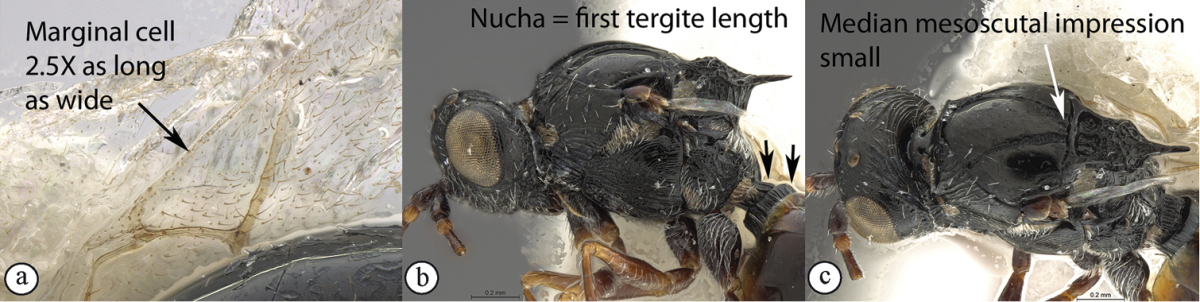	
2	Marginal cell 2.5× as long as wide (a). Nucha short, equivalent in length to first tergite (b). First tergite 0.25× as long as high in lateral view (b). Second flagellar segment longer than first. Median mesoscutal impression small (c)	***Xyalophora provancheri* Jiménez & Pujade-Villar**
	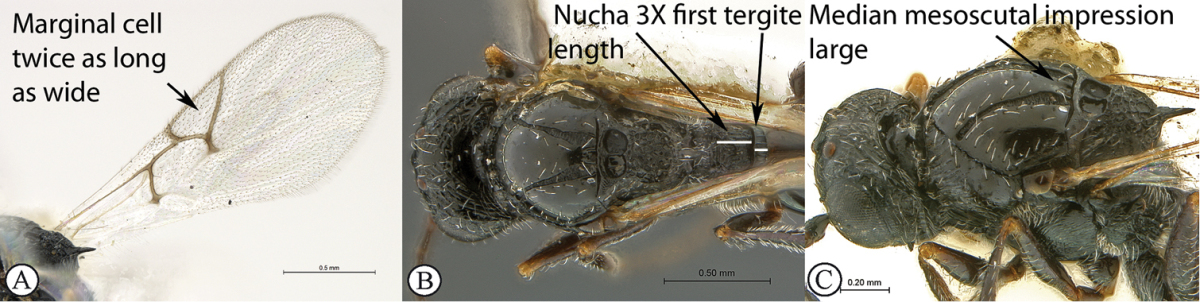	
–	Marginal cell twice as long as wide (A). Nucha long, 3× first tergite length (B). First tergite 0.2× as long as high in lateral view. Second flagellar segment as long as first. Median mesoscutal impression large, distinct (C)	***Xyalophora tintini* sp. n.**

#### Xyalophora provancheri

Taxon classificationAnimaliaHymenopteraFigitidae

Jiménez & Pujade-Villar, 2008

Figures 7 , 8

##### Type material.

**Holotype. Female:** C-335, Burkina Faso, Komprenya, 1–6.VI.1988, Sanborne, Landry & Tou Sarame” (white label); “Holotype desig.-2006 Jiménez & Pujade-Villar” (red label); “*Xyalophora provancheri* sp. n. & Jiménez & Pujade-Villar, det. 2006” (white label); IMAGED WaspWeb LAS 4.4 SAMC 2014 (yellow label). Deposited in CNCI, Ottawa.

##### Diagnosis.

Occipital carinae parallel, fine. Scutellar spine long, 0.5× length of the scutellum (excluding spine). First tergite short (0.25× as long as high in lateral view; equivalent in length to nucha in dorsal view). Notauli widened posteriorly (maximum width 0.8× the minimum distance separating them towards posterior mesoscutal margin). Marginal cell 2.5× as long as wide. Second flagellar segment longer than first.

**Figure 7.**
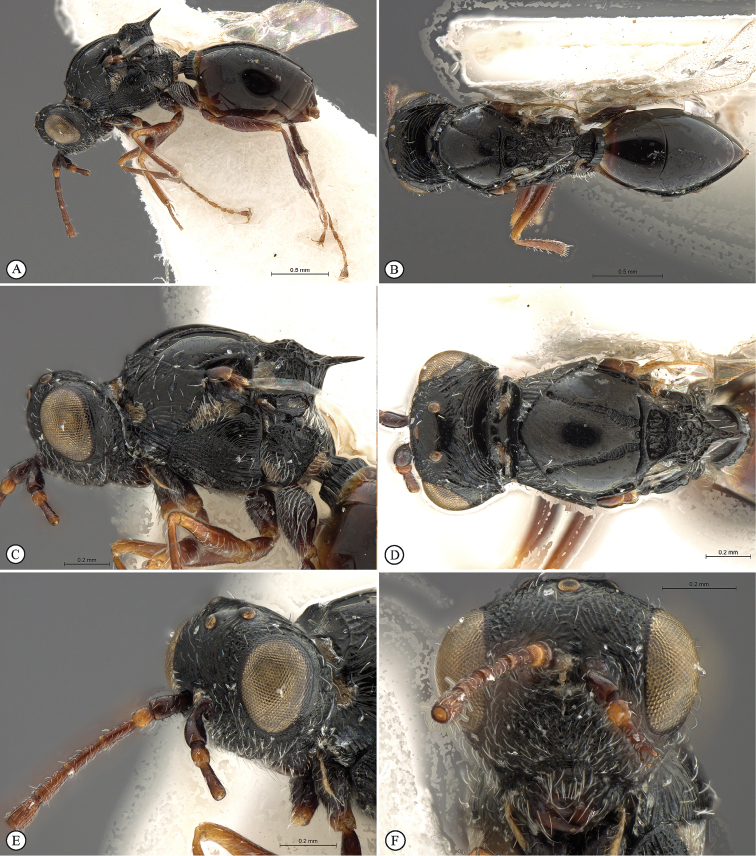
*Xyalophora provancheri*, holotype female. **A** lateral habitus **B** dorsal habitus **C** head and mesosoma, lateral view **D** head and mesosoma, dorsal view **E** head and antennae, anterio-lateral view **F** head, anterior view.

**Figure 8.**
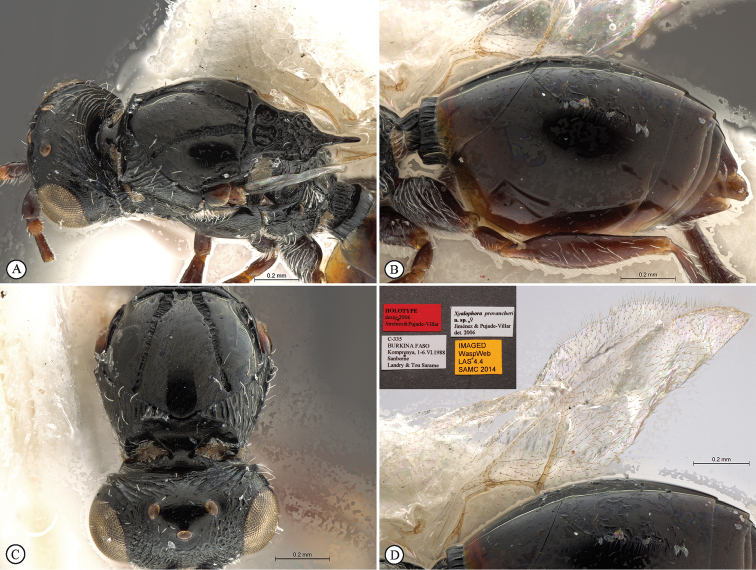
*Xyalophora provancheri*, holotype female. **A** head and mesosoma, dorso-lateral view **B** metasoma, lateral view **C** head and partial mesosoma, anterio-dorsal view **D** forewing (inset: data labels).

##### Distribution.

Burkina Faso.

#### Xyalophora tedjoansi

Taxon classificationAnimaliaHymenopteraFigitidae

van Noort, Buffington & Forshage sp. n.

http://zoobank.org/9C36B235-2E9C-4067-BCA5-05ACFA2F7710

Figures 9 , 10

##### Type material.

**HOLOTYPE.** Female: COLL. MUS. TERVUREN, Mali: Cinzana, 18-ix-1970, G. Pierrard, Imaged WaspWeb SAMC 2012 (yellow label), Holotype F ***Xyalophora tedjoansi*** van Noort, Buffington & Forshage (red label) [point-mounted on white card] (RMCA).

**PARATYPE.** 1F: COLL. MUS. TERVUREN, Mali: R.C T. – M’Pesoba, 11-vii-1970, G. Pierrard, Imaged WaspWeb, SAMC 2012 (yellow label), Paratype F ***Xyalophora tedjoansi*** van Noort, Buffington & Forshage (yellow label) (RMCA).

##### Distribution.

Mali.

##### Etymology.

The specific epithet *tedjoansi* is in the genitive case and is to commemorate the American-cosmopolitan poet Ted Joans (1928–2003), a surrealist, beat, black power and jazz activist who made Mali one of his several homes in the world. The *Xyalophora* spine may suggest the horn of Joans’ totemic rhino.

##### Diagnosis.

The large first tergite (petiole) and infuscate forewings surrounding the venation and radial vein meeting wing margin at almost 90 degrees immediately distinguish this species. Head subquadrate, 1.1× wider than long (*Xyalophora provancheri* distinctly wider than long 1.24×). Occipital carinae parallel, fine as in *Xyalophora provancheri* contrasting with the discontinuous rugose carinae of *Xyalophora tintini*. Antennae clavate as in *Xyalophora provancheri*. Scutellar spine long, 0.8× length of the scutellum (excluding spine). Scutellar spine shorter in the other two Afrotropical species (0.5× length of the scutellum). Petiole (T2) longer than in other two species:1.6× higher than long in lateral view (3.6–4× higher than long in other two species). Notauli narrow, 1/3 of the distance separating them at posterior mesoscutal margin; broader in other two species (1/2 of the distance separating them at posterior mesoscutal margin). Marginal cell venation much thicker (except for thin marginal vein) than in the other two species; Rs radial vein meeting wing margin at almost 90 degrees (Rs meets wing margin at acute angle in *Xyalophora provancheri* and *Xyalophora tintini*). Wings infuscate around venation, hyaline in *Xyalophora tintini*. First tergite (petiole) long (0.6× as long as high in lateral view; twice as long as nucha in dorsal view).

##### Description.

FEMALE. Length 1.85 mm. Head, mesosoma black; metasoma dark brown. Antennae brownish-orange, darkening towards terminal three segments. Legs brownish-orange, except for coxae, which are darker. Wings transparent; with irregular infuscation either side of the basalis vein, and in marginal cell.

*Head*. Head subquadrate, 1.1× wider than long. Entire head, including eyes, with scattered pubescence, pubescence densest on lower face. Eyes not laterally extended, confluent with outer margin of gena in frontal view. Antenna 13 segmented; F1 slightly shorter than F2; flagellum widening gradually toward apex with final segment (F11) globular. Vertex polished, ocellar fig slightly raised; ocelli normal, their diameter half the distance between lateral and median ocellus (COC); POC:OOC:COC = 20:15:12. Upper face coriaceous, antennal scrobes not delimited. Occiput weakly concave in dorsal view, with numerous sub-parallel, occasionally reticulate, carinae radiating from occiptal carinae and directed medially towards ocelli, but terminating well before lateral ocelli. Lower face rugulose, weakly humped between toruli and clypeal margin, slightly protruding in lateral view. Upper clypeal margin defined by pronounced excavation containing anterior tentorial pits. Clypeus with strong medial hump dorsally, concave ventrally with strong pubescence. Gena rugulose in malar space, coriaceous medially and dorsally.

**Figure 9.**
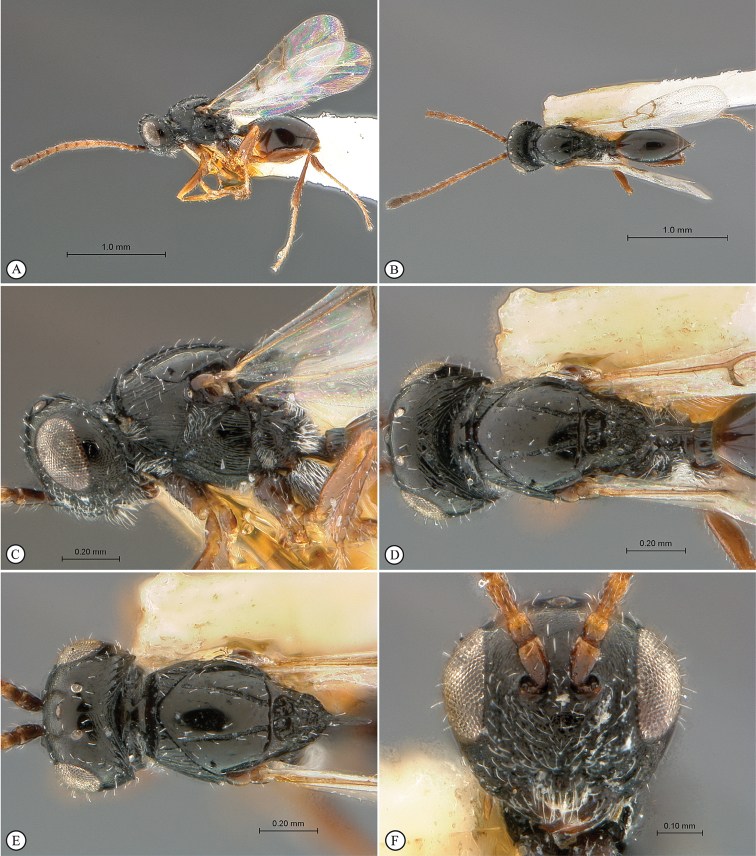
*Xyalophora tedjoansi* sp. n., holotype female. **A** lateral habitus **B** dorsal habitus **C** head and mesosoma, lateral view **D** head and mesosoma, dorsal view **E** head and mesosoma, posterior-dorsal view **F** head, anterior view.

*Mesosoma*. With scattered pubescence. Anterior fig of pronotum polished, glabrous dorsally and medially, setose laterally on bridge; fovea closed with narrow lateral bridge; fig dorsally and laterally defined by strong pronotal carina. Lateral surface of pronotum horizontally striate, anterio-medially and ventrally with patch of dense white setae. Mesoscutum polished with lines of strong white setae each side of notauli and along lateral margins of scutum. Notauli almost complete, terminating just before anterior margin of mesoscutum; transversely striate; only slightly widening towards posterior mesoscutal margin (maximum width 0.35× the minimum distance separating them towards posterior mesoscutal margin); median mesoscutal impression very faint, weakly defined; parascutal impressions defined, sculpturing similar to notauli. Mesoscutum convex and scutellum anteriorly humped in lateral view. Scutellar fovea each with a longitudinal carina. Scutellum strongly areolate-rugose. Scutellar spine elongate, 0.8× scutellar length (excluding spine). Mesopleural triangle defined without ventral carina, strongly pubescent; posterior half (including speculum) of mesopleuron horizontally striate, anterior half rugulose-punctate; mesopleural carina defined. Metepisternum ventrally excavated with pubescence, medially longitudinally striate. Metepimeron depressed with pubescence. Dorsellum laterally strongly excavated. Lateral propodeal carina present. Lateral propodeal area densely pubescent. Rs+M of forewing weakly defined distally at junction with 2r, but otherwise absent. Basalis vein present. M+Cu1 absent. Marginal cell closed, 1.55× as long as wide, veins thick, contrasting with thin marginal vein. Radial vein meets wing margin at almost 90 degrees. Margin with fringe of setae. Legs sparsely punctate, pubescent. Metacoxa stongly and densely pubescent. Mesotibial and metatibial outer spur shorter than inner spur. Ratio of first metatibial segment to the remaining 4 segments: 0.77×.

*Metasoma*. Tergites polished. First tergite longitudinally striate, 4.3× as wide as long in dorsal view; 1.6× higher than long in lateral view; twice as long as nucha in dorsal view. T4 the largest tergite. Relative dorsal length of T3–8: 75:95:15:15:20:15. Posterior margin of T7 evenly curved. T8 exposed. Ovipositor valves not extending beyond apex of metasoma, concealed within T8. Hypopygium not extending beyond T8.

**Figure 10.**
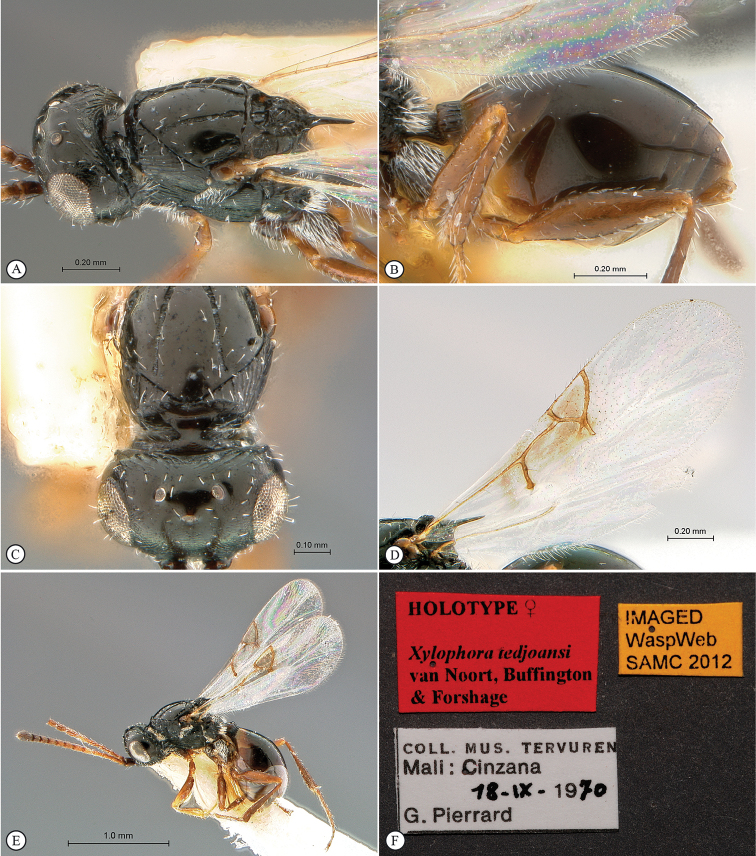
*Xyalophora tedjoansi* sp. n., holotype female. **A** head and mesosoma, dorso-lateral view **B** metasoma, lateral view **C** head and partial mesosoma, anterio-dorsal view **D** forewing and hind wing **E** paratype female, lateral habitus **F** data labels.

#### Xyalophora tintini

Taxon classificationAnimaliaHymenopteraFigitidae

van Noort, Buffington & Forshage sp. n.

http://zoobank.org/B80EB32C-A928-4F39-BD29-3E6D02EBDC1A

Figures 11 , 12

##### Type material.

**HOLOTYPE** Female. **DEMOCRATIC REPUBLIC OF CONGO:** Congo belge: P.N.U. Mabwe (r. E. lac Upemba), (585m) 15-viii-1947. Miss. G.F. de Witte. 678a, Holotype F ***Xyalophora tintini*** van Noort, Buffington & Forshage (red label) [point-mounted on white card] (RMCA).

**PARATYPES. NAMIBIA**, 1 male: South West Africa (W22), Kuiseb river canyon, 22–23.i.1972, Riverside vegetation, Southern African Exp. B.M.1972-1 (BMNH). **SOUTH AFRICA**, 1 female: Cape Province, Swellendam, ii.1932, S. Africa, R.E. Turner, Brit Mus., 1932-145 (BMNH).

##### Diagnosis.

Occiput with parallel carinae (but with some discontinuous reticulation on left hand side of occiput in holotype female). Head distinctly (1.25×) wider than long as in *Xyalophora provancheri* (*Xyalophora tedjoansi* subquadrate 1.1×). Scutellar spine shorter, 0.5× length of the scutellum (excluding spine) as in *Xyalophora provancheri* (longer, 0.8× in *Xyalophora tedjoansi*). Notauli widened posteriorly, narrow in *Xyalophora tedjoansi*. Median mesoscutal impression distinct. Marginal cell 2.0× as long as wide (2.5× in *Xyalophora provancheri*). Wings hyaline, infuscate around venation in *Xyalophora tedjoansi*.

**Figure 11.**
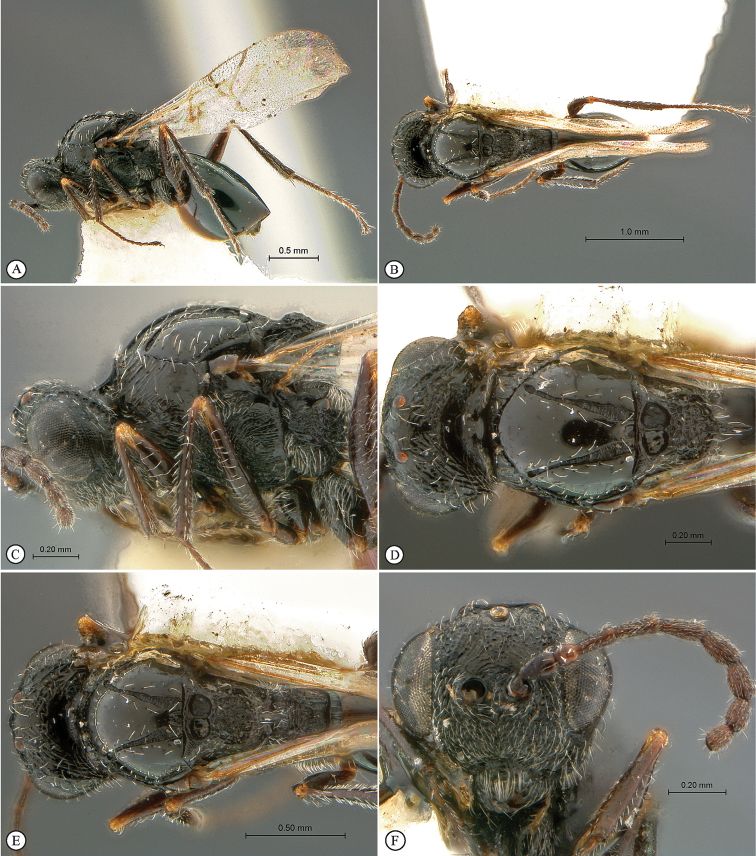
*Xyalophora tintini* sp. n., holotype female. **A** lateral habitus **B** dorsal habitus **C** head and mesosoma, lateral view **D** head and mesosoma, dorsal view **E** head and mesosoma, posterior-dorsal view **F** head, anterior view.

**Figure 12.**
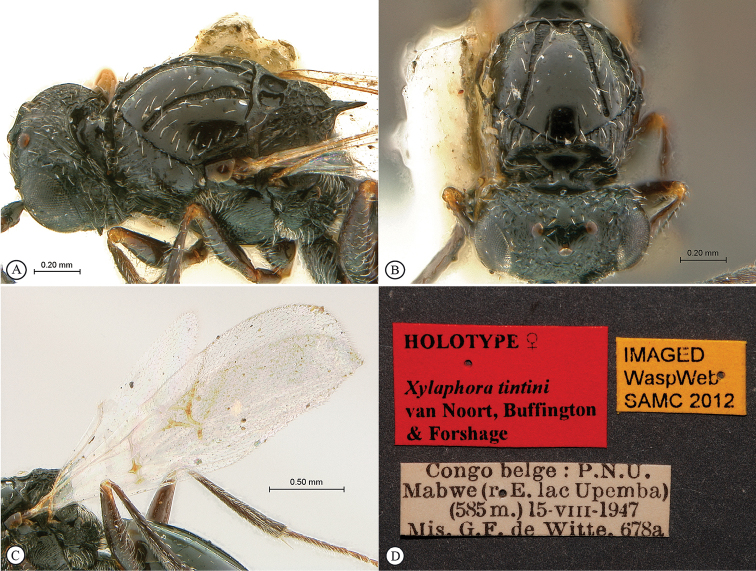
*Xyalophora tintini* sp. n., holotype female. **A** head and mesosoma, dorso-lateral view **B** head and partial mesosoma, anterio-dorsal view **C** forewing and hind wing **D** data labels.

##### Distribution.

Democratic Republic of Congo, Namibia, South Africa.

##### Etymology.

The specific epithet *tintini* is in the genitive case and is for Tintin, the comic book character by Hergé, whose adventures in the Congo have done much to popularise the country in a very controversial manner in parts of the world. The *Xyalophora* spine may possibly suggest Tintin’s famous tuft of hair.

##### Description.

FEMALE. Length 2.75 mm. Head, mesosoma and metasoma black. Antennae and legs dark brown except for coxae, which are black. Wings transparent; without any infuscation.

*Head*. Head transverse. 1.25× wider than long. Entire head, including eyes, with scattered pubescence, pubescence densest on lower face. Eyes not laterally extended, almost confluent with outer margin of gena in frontal view. Antennae damaged in holotype, only 10 segments remaining on left antennae (right antenna missing); F1 equal in length to F2; flagellum widening toward remaining end. Vertex weakly rugulose, polished adjacent to lateral ocelli, ocellar fig slightly raised, polished, weakly laterally defined with confused carinae; lateral ocellus diameter 0.65× the distance between lateral and median ocellus (COC); POC:OOC:COC = 30:20:17. Upper face rugose, antennal scrobes not delimited, but scrobal area with parallel striations directed towards medial rugose area anterior to medial ocellus. Occiput weakly concave in dorsal view, rugulose, with some parallel carinae, medially polished. Lower face rugulose, weakly humped between toruli and clypeal margin, slightly protruding in lateral view. Upper clypeal margin defined by pronounced excavation containing anterior tentorial pits. Clypeus with strong medial longitudinal hump dorsally, concave ventrally with strong, long pubescence; clypeal margin evenly convex. Gena finely colliculate in malar space, coriaceous medially and dorsally; genal carina defined with strong linear alveolations.

*Mesosoma*. With scattered strong pubescence. Anterior fig of pronotum polished, glabrous dorsally and medially, setose laterally on bridge; fovea closed with broad lateral bridge; fig dorsally and laterally defined by strong pronotal carina. Lateral surface of pronotum horizontally striate, anterio-medially and ventrally with patch of white setae. Mesoscutum polished with lines of strong white setae each side of notauli and along lateral margins of scutum. Notauli almost complete, terminating just before anterior margin of mesocutum; transversely striate; posteriorly broadening abruptly prior to narrowing again at posterior mesoscutal margin; widened posteriorly (maximum width 0.7× the minimum distance separating them towards posterior mesoscutal margin); median mesoscutal impression present as small circular concavity; parascutal impressions defined, sculpturing similar to notauli. Mesoscutum convex and scutellum anteriorly humped in lateral view. Scutellar fovea each with an incomplete longitudinal carina. Scutellum strongly areolate-rugose. Scutellar spine elongate, 0.5× scutellar length (excluding spine). Mesopleural triangle defined without ventral carina, strongly pubescent; posterior half (except for polished speculum) of mesopleuron horizontally striate, anterior half rugulose-punctate; mesopleural carina defined. Metepisternum ventrally excavated with pubescence, medially rugulose. Metepimeron depressed with pubescence. Dorsellum laterally strongly excavated. Lateral propodeal carina present. Lateral propodeal area densely pubescent. Rs+M of forewing weakly defined distally at junction with 2r, but otherwise absent. Basalis vein present. M+Cu1 absent. Marginal cell 2.0× as long as wide, closed along wing margin with weak vein. Margin with fringe of setae. Legs sparsely punctate, pubescent. Metacoxa stongly and densely pubescent. Mesotibial and metatibial outer spur shorter than inner spur. Ratio of first metatibial segment to the remaining 4 segments: 0.88×.

*Metasoma*. Tergites polished. First tergite (petiole) short, longitudinally striate, 3.5× as wide as long in dorsal view; 5× higher than long in lateral view; a third of nucha length in dorsal view. T4 the largest tergite. Relative dorsal length of T3–T4: 7:10; T5–T8 hidden beneath T4. Ovipositor valves not extending beyond apex of metasoma, concealed within T8. Hypopygium not extending beyond T8.

MALE. Length 2 mm. Head, mesosoma and metasoma black. Antennae reddish-brown; legs brown except for coxae, which are dark brown and femur/tibial junctions which are paler. Wings transparent; without any infuscation.

*Head*. As in female, except for antenna with 14 segments: F1 0.85× length of F2; F3-F7 equivalent in length to F2. F8–F11 equivalent in length to F1; ultimate segment 1.65× F1; and occiput with parallel semi-reticulate carina, medially polished.

*Mesosoma* as in female, except for stronger forewing venation and slightly more hirsute wing surface and fringe.

*Metasoma*. Tergites polished. First tergite (petiole) short, polished with isolated longitudinal striae, 3× as wide as long in dorsal view; equivalent to nucha length in dorsal view. T4 the largest tergite. Relative dorsal length of T3–T5: 7:9:1; T6–T7 hidden beneath T5.

##### Comments.

The much smaller second female from South Africa has a very thin, polished second tergite, with no discernable longitudinal striations. Possibly these are hidden under the overlapping anterior margin of the third tergite. Otherwise this specimen keys to *Xyalophora tintini*. This species is very similar to *Xyalophora provancheri* only being separated by the shorter first tergite and slightly broader marginal cell. The tendency towards rugulose sculpture on the occiput of the holotype specimen is likely to be related to the larger size of the holotype and not diagnostically useful.

## Conclusion

Representatives of Afrotropical Figitinae are rare in collections and the available specimens probably represent a superficial gathering of actual species richness. Very few countries are represented in the material that was available for examination and with further sampling, potentially using less commonly deployed collecting techniques such as carrion traps, fecal traps and emergence traps, many more specimens are anticipated. However, from experience with Malaise trapping projects in central, east and southern Africa, Afrotropical Figitidae may be locally abundant, and if the trap is in the wrong place at the wrong time, the wasps will be missed. These projects, which have resulted in months of Malaise trap samples, yielded precious few Figitinae. In contrast, Malaise trapping programs in North, Central and South America have yielded many specimens and species of Figitinae, but these samples emanate from traps sited in close association with areas of human disturbance and/or domesticated livestock. Since core figitines are associated with brachyceran flies, it is logical that we would find these wasps where we find the flies, and many of these flies are associated with homo-specific environments. Perhaps future collecting in the Afrotropical Region, in or near homo-specific environments, will yield hitherto unknown species of Figitinae.

## Supplementary Material

XML Treatment for Figites

XML Treatment for Figites aciculatus

XML Treatment for Lonchidia

XML Treatment for Lonchidia clavicornis

XML Treatment for Neralsia

XML Treatment for Neralsia haddocki

XML Treatment for Xyalophora

XML Treatment for Xyalophora provancheri

XML Treatment for Xyalophora tedjoansi

XML Treatment for Xyalophora tintini
